# Challenges of microtome‐based serial block‐face scanning electron microscopy in neuroscience

**DOI:** 10.1111/jmi.12244

**Published:** 2015-04-23

**Authors:** A. A. WANNER, M. A. KIRSCHMANN, C. GENOUD

**Affiliations:** ^1^ Friedrich Miescher Institute for Biomedical ResearchMaulbeerstrasse 664058BaselSwitzerland

**Keywords:** Microtome, registration, SBFSEM, serial block‐face scanning electron microscopy (SBEM), segmentation, three‐dimensional reconstruction, tiling

## Abstract

Serial block‐face scanning electron microscopy (SBEM) is becoming increasingly popular for a wide range of applications in many disciplines from biology to material sciences. This review focuses on applications for circuit reconstruction in neuroscience, which is one of the major driving forces advancing SBEM. Neuronal circuit reconstruction poses exceptional challenges to volume EM in terms of resolution, field of view, acquisition time and sample preparation. Mapping the connections between neurons in the brain is crucial for understanding information flow and information processing in the brain. However, information on the connectivity between hundreds or even thousands of neurons densely packed in neuronal microcircuits is still largely missing. Volume EM techniques such as serial section TEM, automated tape‐collecting ultramicrotome, focused ion‐beam scanning electron microscopy and SBEM (microtome serial block‐face scanning electron microscopy) are the techniques that provide sufficient resolution to resolve ultrastructural details such as synapses and provides sufficient field of view for dense reconstruction of neuronal circuits. While volume EM techniques are advancing, they are generating large data sets on the terabyte scale that require new image processing workflows and analysis tools. In this review, we present the recent advances in SBEM for circuit reconstruction in neuroscience and an overview of existing image processing and analysis pipelines.

## Lay Description

During the last decades, numerous technologies have been developed in order to visualize the brain at high resolution with Volume Electron Microscopy. The visualization in three dimensions and the analysis of an entire brain from an animal model where all cells are visible and can be followed and where all synapses can be examined and counted is considering as a milestone by numerous neuroscientists. One of the technique that could be used to acquire images of an entire brain at nanometre scale is the microtome‐based block‐face scanning electron microscopy developed by W. Denk and his collaborators. In this approach, a microtome integrated within the specimen chamber of a scanning electron microscope cuts away nanometres thin slice of the specimen, thus allowing the imaging of the next ‘layer’. These individual 2D images are then assembled to a 3D image of all neurons and their projections. The recent improvements of the technique due to better microscopes and better sample preparation are pushing the limits of data quality and acquisition speed. It is creating data sets requiring important storage space. At the level of analysis, new algorithms are developed to reconstruct manually or with semiautomated interfaces all the neurons present in a volume.

## Volume EM techniques

Understanding the information flow in neuronal circuits of the brain requires a detailed map on the connectivity between neurons. In order to map all the connections of even a small circuit, detailed recognition of anatomical structures such as spine necks and synapses is necessary. It requires 3D ultrastructural resolution on the nanometre scale (<20 nm pixel^−1^; Helmstaedter, 2013). In addition, neurons are densely packed and a cubic millimetre, the typical size of a voxel in magnetic resonance imaging, contains millions of neurons and thousands of metres neurite length (Behrens *et al*., 2007). Therefore, microscopy techniques that rely on sparse labelling of neuronal features, such as fluorescence microscopy, are not suited for dense circuit reconstruction. The commonly used techniques for dense circuit reconstructions are volume EM techniques such as serial section TEM (ssTEM), automated tape‐collecting ultramicrotome SEM (ATUM‐SEM), serial block‐face SEM (SBEM) and focused ion beam SEM (FIB‐SEM). The former two use nondestructive manual (ssTEM) or automated (ATUM) ultrathin sectioning and slice collection with subsequent imaging. The latter two use *in situ* destructive on‐block sectioning inside of the SEM vacuum chamber either by a diamond knife (SBEM) or with a focused ion beam (FIB‐SEM).

In ssTEM, sections are cut by hand using an ultramicrotome and collected onto grids. Sections are imaged in a TEM, and imaging approaches such as camera array imaging (TEMCA) with typically 2–4 nm lateral resolution (Bock *et al*., 2011; Takemura *et al*., 2013; Atasoy *et al*., 2014) can acquire images at a rate of 5–8 megapixels s^−1^ (Bock *et al*., 2011). However, the manual sectioning process is prone to errors and is also labour‐intensive. It limits the typical section thickness to 40–50 nm and the number of consecutive sections to a few thousands, because sections can get folded or warped or even get lost. Furthermore, the subsequent registration step is more delicate due to distortions of sections occurring during cutting and imaging (Kaynig, Fischer, *et al*., 2010; Saalfeld *et al*., 2010).

ATUM‐SEM (Schalek *et al*., 2011) overcomes these problems partially by automated cutting and collection of sections on an electrically opaque tape. This allows to reliable cut thousands of subsequent sections as thin as 30 nm. The on‐tape sections are further processed for storage on silicon wafers and subsequent imaging in an SEM (Hayworth *et al*., 2014).

In SBEM [formerly called SBFSEM but renamed by W. Denk (Denk & Horstmann, 2004)], the recording chamber of an SEM is equipped with a microtome. After each cut, the block‐face is imaged with the scanning beam before the next section is shaved off. SBEM achieves field of views of >0.5 × 0.5 mm^2^ at a lateral resolution on the order of 6–10 nm and reliable cuts thousands of sections at section thickness 20–30 nm for neural tissue at an acquisition rate of 0.5 to 2 megapixel s^−1^ (Briggman *et al*., 2011; Helmstaedter, 2013). In FIBSEM, slices are cut using a gallium‐ion beam (Knott *et al*., 2008). This allows to cut sections as thin as 5 nm with a lateral resolution <5 nm and an acquisition rate of 0.1–0.5 megapixel s^−1^ (Knott *et al*., 2008; Boergens & Denk, 2013; Maco *et al*., 2014). However, the field of view is limited to <0.1 × 0.1 mm^2^.

The major advantage of the destructive on‐block methods is that they do not suffer from warping problems and section loss that can significantly affect the data quality and the subsequent data analysis. In contrast, the advantages of the nondestructive methods are that sections can be imaged multiple times (e.g. at different magnifications) and in parallel on multiple microscopes, which reduce the acquisition time significantly compared to sequential techniques such as SBEM and FIBSEM.

Currently, these techniques are to a large degree complementary. They cover different range of resolution, field of view and automation (Lichtman & Denk, 2011).

To date, only a handful of neuronal circuits have been completely reconstructed using volume EM. The entire nervous system of the nematode Caenorhabditis elegans has been acquired using serial sections and TEM, which led to the first complete connectome of an entire organism (White *et al*., 1986; Varshney *et al*., 2011). It includes all 302 neurons connected by more than 7000 synapses. Partial connectomes have been obtained from a mouse retina (Briggman *et al*., 2011; Helmstaedter *et al*., 2013) by SBEM and from mouse primary visual cortex (Bock *et al*., 2011) and Drosophila visual motion detection circuit (Takemura *et al*., 2013) by ssTEM. The retina datasets are available on http://www.knossostool.org/ and Bock *et al*.'s images are publicly available through the Open Connectome Project (http://www.openconnectomeproject.org/).

## Development of SBEM for connectomics

SBEM was first described in 2004 (Denk & Horstmann, 2004) based on an idea introduced by Leighton (1981). The group of W. Denk inserted a completely redesigned ultramicrotome inside of an environmental scanning electron microscope with a field emission gun. In this design, SBEM allows for automated, iterative removal of thin sections from the sample block and subsequent block‐face imaging at ultrastructural resolution. The ultramicrotome comes with a movable high‐precision stage that allows dividing the field of view into a mosaic of tiled images. This allows, in principle, to acquire field of views of arbitrary size, although the edge size of a single image in an SBEM is currently limited to about 40 μm (3VBSED, Gatan Inc.).

One cubic millimetre is often described as the minimal target volume for neuronal circuit reconstruction, because it corresponds to a volume large enough to analyze a functional unit such as a cortical column in the adult mouse brain. For the dense reconstruction of all neurites and the unambiguous identification of chemical synapses in this volume, a voxel size of about 10 × 10 × 30 nm is necessary. This volume corresponds to a data set of 3.333 × 10^14^ voxels requiring >300 TB storage space. At a typical acquisition speed of 1–4 microseconds pixel^−1^, the cubic millimetre requires more than 18 years of acquisition time making this kind of project impossible. Thereby, the stage movements for the tiling alone causes an overhead of 4 years.

In order to move this kind of projects within the realm of the feasible, both, the instrumentation and the sample preparation, have been improved and optimized in the last couple of years.

At the level of instrumentation, progress has been made that now allows now to scan larger field of views with higher scan speed and lower beam voltage. The neuronal tissue used for volume EM is usually embedded in nonconductive resin. If nonconductive regions are scanned in an SBEM, electrons accumulate on the block surface and cause charging artefacts. Therefore, environmental SEMs have been used for SBEM, where a gaseous agent such as water or nitrogen (Danilatos, 1988; Danilatos, 2009; Danilatos, 2012) is introduced into the recording chamber and takes up exceeding electrons from the block‐face. This reduces any charging artefacts significantly (Denk & Horstmann, 2004), but requires a lower scanning speed and/or a higher beam voltage in order to compensate for the loss in signal due to electron scattering at the gaseous agent. Because the beam current (number of electrons) of these environmental SEMs is limited to approximately 100 pA, the scan speed/dwell time typically had to be at least around 1–5 microseconds pixel^−1^ in order to collect enough electrons for a sufficient signal‐to‐noise ratio (Briggman *et al*., 2011). Similarly, for a decent image quality, the beam voltage (speed of the beam electrons) on these instruments had to be larger than 2 kV. However, the higher the beam voltage, the deeper the electrons penetrate into the block‐face, which decreases the Z resolution and increases the minimal section thickness that can be cut (Denk & Horstmann, 2004). In order to reliably cut 25–30‐nm thin sections, a beam voltage <2 kV is necessary. Therefore, new SEMs are optimized for low voltage (1.5–2 kV) and higher beam currents (>0.5 nA) that allow faster scanning and shorter dwell times (<1 microsecond pixel^−1^; Titze & Denk, 2013).

Recently, new approaches have been proposed to overcome the block‐face charging problem even in high‐vacuum operation mode of an SEM. A coating device can be introduced into the SEM chamber sputtering a thin conductive layer after each cut before imaging which makes the block‐face perfectly conductive (Titze & Denk, 2013). As introduced above, the stage movements for the acquisition of a tiled field view can cause a significant overhead of more than 20% of the total acquisition time. Therefore, the groups of Winfried Denk (MPIN Munich), Moritz Helmstaedter (MPI Frankfurt) and Kevin Briggman (NIH, Circuit Dynamics and Connectivity Unit, Bethesda) developed new microtomes that allow to continuously move the sample stage along one axis while scanning, which minimizes the number of stage movements for tiled acquisition and results in a significantly reduction of acquisition time (Perkel, 2014; http://www.pbs.org/newshour/updates/watch‐ideas‐light‐fishs‐brain/).

Another promising approach to reduce the number of image tiles is the new multiSEM 505, launched by ZEISS (Marx, 2013). It allows scanning sample surfaces or sections with 61 parallel electron beams simultaneously, which increases the field of view tremendously. Combined with ATUM‐SEM or with block‐face approaches, it will allow to image at a rate of 1220 megapixels s^−1^ (Eberle *et al*., 2015).

Currently, Winfried Denk is pushing the extremes of SBEM with the goal of cutting and imaging an entire mouse brain (Mikula *et al*., 2012; Perkel, 2014). Besides the development of a completely new microtome capable of cutting centimetre‐sized blocks, they are also working on improving the stability of image acquisition by developing methods for automated correction of focus and stigmatism (Binding *et al*., 2013).

At the level of the sample preparation, several groups have put efforts in generating samples able to sustain the accelerating voltage and beam current necessary for high‐speed acquisition while keeping signal‐to‐noise ratio sufficient for subsequent analysis, such as image segmentation. The staining protocols have been adapted and optimized for SBEM (Deerinck *et al*., 2010; Mikula *et al*., 2012; Tapia *et al*., 2012; Starborg *et al*., 2013) focusing on the *en bloc* staining with diverse heavy metals: OTO (osmium‐thiocarbohydrazide‐osmium, rOTO (reduced Osmium), OTrO followed by *en bloc* uranyl acetate and/or lead citrate/asparte) in order to increase the contrast and the conductivity of the sample. The latter also reduces charging issues during acquisition. However, samples stained by multiple steps of heavy metals show reduced permeability and resin may not be able to fully penetrate (Mikula *et al*., 2012; Tapia *et al*., 2012; Starborg *et al*., 2013). In addition, the density of heavy metals in the sample block can compromise the cutting properties such as slice thickness and may reduce the imaging quality.

The advances on both levels, sample preparation and instrumentation, set new conditions of imaging: With higher beam currents (>1 nA) scan speed can reach 10 MHz (100 nanoseconds pixel^−1^) without charging artefact, while keeping the signal‐to‐noise ratio good enough for image segmentation and neurite reconstruction (Titze & Denk, 2013). At a speed of 10 MHz (0.1 microsecond pixel^−1^) and with continuous scanning along one axis, the millimetre cube could be acquired in about 1.5 year. This is still a significant challenge as these instruments currently cannot be run for more than 1–2 months (Briggman *et al*., 2011; Lichtman & Denk, 2011; Helmstaedter *et al*., 2013) without failure. However, this calculation is based on a single‐beam SEM. With the launch of the multi‐SEM 505, the acquisition can be parallelized on 61 beams, leading to a speed of 1220 megapixels s^−1^ (vs. 1 megapixel s^−1^ for a single‐beam SEM at 1 microsecond pixel^−1^ equivalent to 1 MHz). With this, the cubic millimetre could be acquired in about 2 months.

Another imaging parameter that influences the effective acquisition time significantly is the size of the acquired field of view. It is therefore advisable to restrict the acquisition to the desired region of interest. However, finding a particular region of interest in an *en bloc* stained sample is challenging. Possible solutions are targeted near‐infrared branding of a region of interest (Bishop *et al*., 2011; Maco *et al*., 2013; Maco *et al*., 2014) and staining specific structures, cells or molecules using genetic labels that accumulate electrodense molecules. (Martell *et al*., 2012).

## Data preprocessing: alignment in 3D

**Figure 1:**
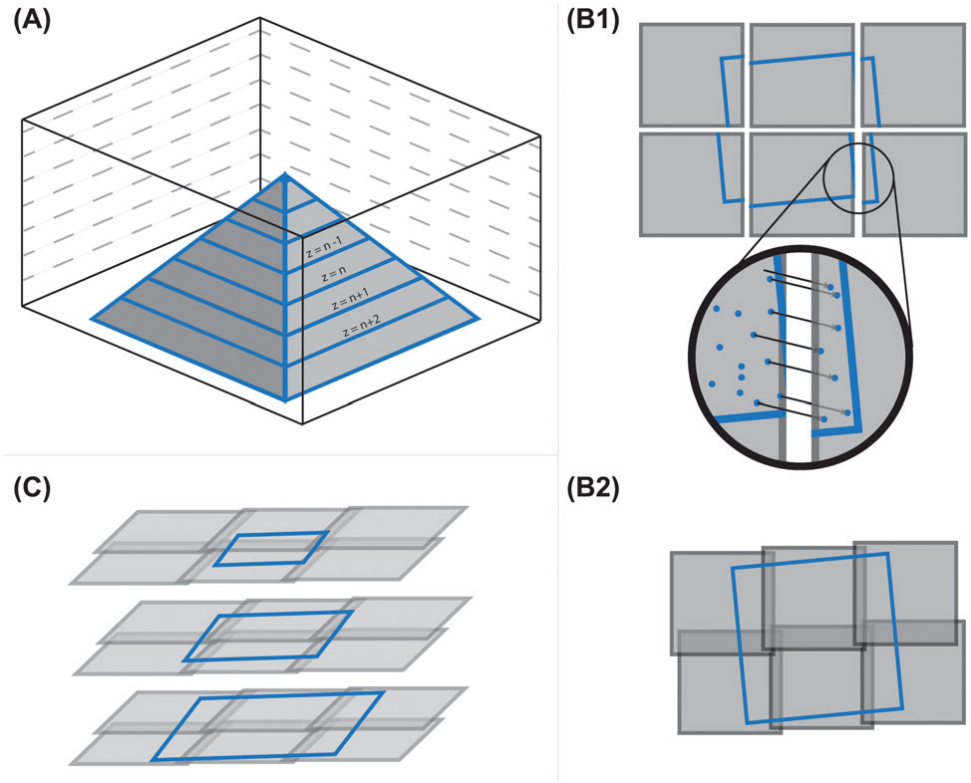
(A) Resin‐embedded specimen (pyramid) is sectioned from top to bottom, while each block‐face is imaged as a mosaic of overlapping tiles (B) For each imaged block‐face, the overlapping tiles taken from the same area are compared in order to determine the correct overlap (C) The mosaics of adjacent planes are compared based on image features (as in B) and shifted to form a continuous representation of the specimen (D) Tiles are stitched into a mosaic optimizing the global overlap for each section.

To annotate/reconstruct the imaged sample *in silico*, image preprocessing is necessary. In particular, images need to be placed correctly in three dimensions to combine the slice data into a volume representing the specimen. Image tiles acquired from the same block‐face need to be stitched together in *x* and *y*. The overlapping region between neighbouring tiles is scanned multiple times, which increases acquisition time and in addition can compromise the cutting quality and cutting thickness due to multiple beam exposure. It is therefore advisable to minimize both, the number of image tiles as well as the amount of overlap between neighbouring image tiles. A compromise in the amount of overlap has to be found because the overlaps need to be large enough to allow for correct image registration and stitching. Because temperature changes during acquisition can cause drifts of the stage relative to the beam source on the order of several nanometres (Boergens & Denk, 2013), one cannot rely on stage coordinates alone. In addition, the actual image positions can also drift due to local changes in the electric field due to charging artefacts. In the case of SBEM, these drifts are mostly limited to translations. The necessary corrections do not need to take into account rotations or nonlinear transforms. To stitch the overlapping images pixel‐perfect, the amount of overlap between adjacent images can be calculated via cross‐correlations or by detecting and comparing matching configurations of image features, as with the scale‐invariant feature transform algorithm (Lowe, 2004). Then, the images are stitched to the resulting mosaic by overlapping the tiles and minimizing the global error at the overlaps (Fig. 1). Subsequently, the stitched mosaics need to be aligned in the *z*‐direction. Here, image feature‐based algorithms suppress overfitting of larger objects which are oriented nonperpendicular to the cutting plane, an artefact typical for cross‐correlation‐based alignments. Since EM images of most biological samples contain many objects at smaller scales than the diagonally cut object, their resulting image features do not have a preferential direction and outnumber the features stemming from the larger object.

The workflow of stitching and aligning needs to be automated to cope with many thousands of images per data set to reduce manual labour. TrakEM2, a free open‐source software specifically designed for reconstruction of neural circuits from terabyte EM data sets is a handy tool for this work flow (Cardona *et al*., 2012). It uses pyramidal data organization to minimize the RAM consumption and necessary data throughput rates of mass storage.

## Image analysis, annotation and segmentation

**Figure 2:**
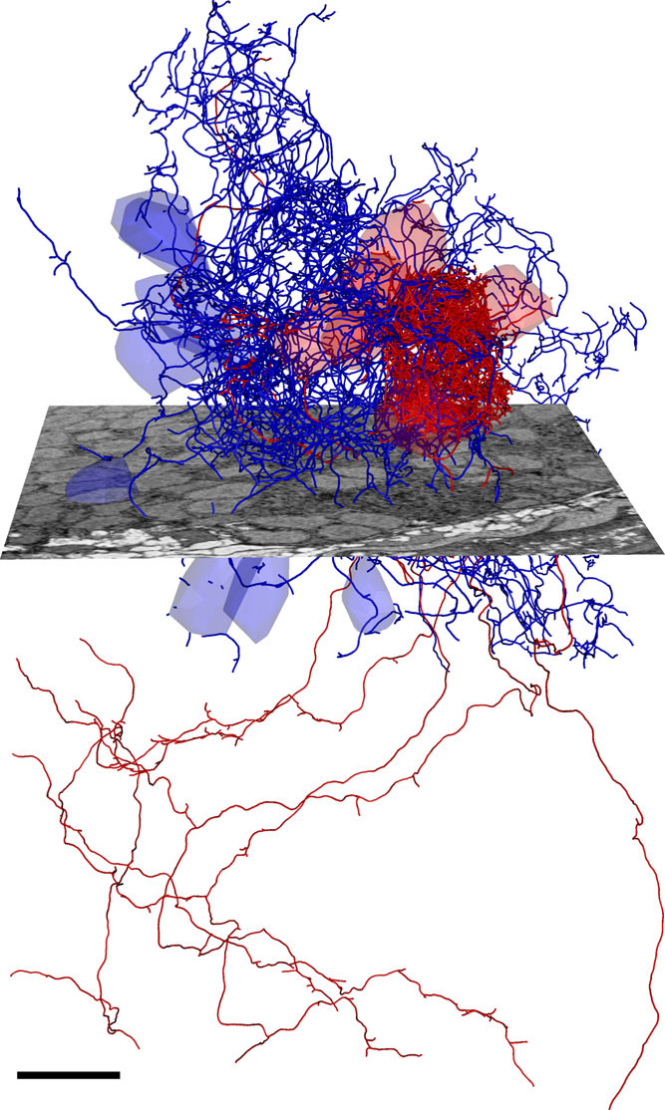
Consolidated skeletons of six mitral cells (red) and eight interneurons (blue) projecting into the same protoglomerulus of the olfactory bulb of a larval zebra fish. The consolidated skeletons were calculated from redundant manual reconstructions of three different tracers. The SBEM data set was acquired in a period of 7 weeks and consists of more than 5000 sections of 25 nm thickness with a lateral pixel size of 10 nm. Scale bar: 10 μm.

Browsing, analyzing and annotating the massive image data sets generated by SBEM is challenging. The retina stack of Briggman *et al*. (2011) and Helmstaedter *et al*. (2013) already required several hundreds of GB storage space, but as the acquisition technology of SBEM progresses, the data set sizes also increase. The acquisition of a cubic millimetre cortex at a voxel size of 10 × 10 × 30 nm would require more than 300 TB storage space after image preprocessing. Therefore, the vast size of the image data makes it impossible to load the full data set for analysis into the RAM of a normal lab computer. Therefore, several labs have developed open‐source software solutions dedicated for large‐scale 3D image data such as KNOSSOS (Helmstaedter *et al*., 2013), TrakEM2 (Cardona *et al*., 2012) and CATMAID (Saalfeld *et al*., 2009). These programs use demand‐driven dynamic data loading procedures, in which only the currently viewed subvolume is loaded into memory. As the user browses through the data, the corresponding subvolumes are continuously loaded in the background and therefore allow seamless navigation with minimal memory requirements. As the data sets are getting too big for local storage, both, CATMAID and KNOSSOS, feature online streaming of the data from external servers. In addition, these programs feature a wide range of manual annotation tools, including feature labelling (e.g. mitochondria, synapses, etc.), skeleton tracing of neurites and volume segmentation (Figure 2).

However, manual annotation and segmentation of large image data sets is tedious, error‐prone and can be very time‐consuming. For example, the dense skeleton reconstruction and analysis of 950 neurons in the inner plexiform layer of a mouse retina required almost 30,000 human working hours (Perkel, 2014), despite 50‐fold speed‐up for manual skeletonization versus manual volume annotation (Helmstaedter *et al*., 2013). Therefore, new software is under development for computer‐assisted, semiautomated large‐scale annotation and segmentation (Lowe, 2004; Jain *et al*., 2007; Chklovskii *et al*., 2010; Kaynig, Fuchs, *et al*., 2010; Kim *et al*., 2014; Maco *et al*., 2014).

Although the currently existing automated segmentation algorithms are still far from perfect, they have been successfully combined with manual annotation or proof‐reading by humans. CATMAID is used for collaborative annotation efforts distributed over collaborating research groups (Saalfeld *et al*., 2009). The SBEM pioneers in the Denk lab recruited hundreds of undergraduates for skeleton tracing of neurons using KNOSSOS (Helmstaedter *et al*., 2013). Thereby, most neurons have been redundantly traced by multiple students in order to form a consensus and in turn reduce reconstruction error rates. Subsequently, these consensus‐skeletons have been used for automated volume segmentation of the corresponding neurons. EyeWire, a crowd sourcing online platform, has successfully used the judgment of thousands of laymen volunteers for the proof‐reading of automatically segmented neurons (Kim *et al*., 2014). Others have employed a small number (<10) of professional proof‐readers (Takemura *et al*., 2013).

## Conclusion

Connectomics, the complete reconstruction of neuronal circuits in different animal models, is a major driving force for new developments in SBEM. New sample preparation protocols, microscopes, acquisition modes and microtomes enable faster acquisition of larger field of views. This in turn creates an unprecedented flood of EM data that triggers the development of new image processing and analysis workflows and software solutions. These technological advances will be and are highly beneficial for many other fields of research.
